# Apigenin Protects H9c2 Cells Against Oxygen–Glucose Deprivation/Reperfusion Injury by Regulating Autophagy via the HIF‐1α/miR‐20a Axis

**DOI:** 10.1002/prp2.70224

**Published:** 2026-03-30

**Authors:** Xiaoxu Zhao, Huihui Li, Yi Yuan, Qingwen Cao, Bo Yu, Wenshu Xue, Gongping Yu, Yao Wang, Xiulong Niu, Yue Wang

**Affiliations:** ^1^ School of Integrative Medicine Tianjin University of Traditional Chinese Medicine Tianjin China; ^2^ Department of Cardiac Intensive Care Medicine Characteristic Medical Center of PAP Tianjin China; ^3^ Aksu Region Uyghur Medical Hospital Aksu, Xinjiang China; ^4^ Department of Cardiology, Tianjin Union Medical Center Nankai University Affiliated Hospital Tianjin China; ^5^ Department of Prevention and Therapy of Cardiovascular Diseases in Alpine Environment of Plateau Characteristic Medical Center of PAP Tianjin China

**Keywords:** apigenin, autophagy, hypoxia inducible factor‐1α, ischemia–reperfusion injury of the infarct myocardium

## Abstract

As living standards improve and the population ages, cardiovascular disease poses a significant threat to human health. Apigenin, a flavonoid compound found in many fruits and vegetables native to warm climates, is named after celery because it is found in the highest concentrations in this plant. Apigenin exhibits various physiological and pharmacological activities. However, its protective effects on the cardiovascular system and the underlying mechanisms are unclear. The aim of this study is to investigate the role of apigenin and its mechanism in protecting against myocardial cell damage induced by OGD/R in H9c2 rat myocardial cells. MTT experiments demonstrated that apigenin protects cells from OGD/R‐induced damage under OGD/R conditions. Molecular mechanism studies showed that apigenin (API) inhibits HIF‐1α protein expression and promotes miR‐20a expression under OGD/R conditions. This reduces the expression of autophagy‐related proteins, inhibits cellular autophagy, and SOD activity. Thus, API exerts a protective effect on cells under OGD/R conditions. These results suggest that apigenin protects cells from oxidative stress damage by inhibiting HIF‐1α expression and promoting miR‐20a expression, thereby reducing autophagy levels in H9c2 cells under OGD/R conditions.

## 1 Introduction

Coronary artery reperfusion is the most effective way to save the ischemic myocardium, protect myocardial function, and prevent patient deaths after acute myocardial infarction (AMI) [1, 2]. However, recanalization inevitably may cause myocardial ischemia–reperfusion injury (MIRI) and damage patients' health [3, 4]. The World Health Organization reports that approximately 7 million people worldwide experience acute myocardial infarction annually, making it the leading cause of death. Currently, there is a lack of effective therapy to prevent MIRI.

MIRI is associated with several factors including calcium overload, inflammatory reactions, increased production of oxygen free radicals, apoptosis, and autophagy [5, 6]. Autophagy has been reported to play a key role in MIRI for its bidirectional regulating effects [7]. Under normal circumstances, moderate autophagy maintains the heart's physical function as a protective factor. In pathological conditions, insufficient or excessive autophagy could lead to metabolite accumulation or autophagy apoptosis [8]. During the early stages of MIRI, autophagy can decompose long‐lived proteins and damaged organelles, which can then be reused by cells to promote cell survival [9]. However, during the reperfusion period, the separation of protein Bcl‐2 from Beclin‐1 can lead to overexpression of autophagy, resulting in cell apoptosis and death [10]. Therefore, regulating autophagy may be considered as a potential strategy for MIRI. However, whether and how autophagy influences the MIRI metabolism remains unclear.

Recently, increasing evidence suggests that non‐coding small RNAs (miRNAs) act as significant mediators in MIRI by regulating autophagy [11, 12]. MiRNAs are short, non‐protein‐encoded RNAs, approximately 22 nucleotides in length. They trigger the degradation or translation inhibition of target mRNA through partial or full base pairing with complementary sites, and negatively regulate the expression of specific genes [13]. A study reported that miR‐20a, an important member of the miR‐17‐92 gene cluster, can activate pathways related to autophagy by regulating different downstream target genes, and then modulate disease progressions such as tumors and MIRI [12]. Xin et al. reported that the level of miR‐20a was down‐regulated in cardiomyocytes treated with H/R, whereas the up‐regulation of miR‐20a could improve cardiomyocyte viability and reduce apoptosis [14]. Therefore, miR‐20a may be a mediator between autophagy and MIRI.

The hypoxia‐inducible factor (HIF) is a heterodimer consisting of the inducible α subunit (HIF‐1α and HIF‐2α) and the constitutively expressed β subunit (HIF‐β/arnt) [15]. As a transcription factor that binds to hypoxia response elements, HIF is responsible for inducing hypoxia genes and repairing the intracellular microenvironment [16]. Several studies reported that HIF‐1α could prevent MIRI and has a cardioprotective effect [17, 18]. HIF‐1α mediates the production of reactive oxygen species through mitochondria and the Nox family, and regulates the redox state of cells, thereby reducing the severity of MIRI [19]. Nevertheless, other reports demonstrated that the polyol pathway in MIRI increased the ratio of NADH/NAD+ in the cytoplasm followed by the HIF‐1α activation and transferrin receptor up‐regulation. This aggravated oxidative damage and increased lipid peroxidation [20]. Further research is needed to determine the dynamic regulation of HIF‐1α on different types of redox indicators at different time points of MIRI. Sun et al. demonstrated that HIF‐1α could bind to the promoter region of miR‐20a and down‐regulate its expression [21]. Moreover, it has been discovered that HIF‐1α can activate BNIP‐3 and NIX, which then combine with Bcl‐2, releasing Bcl‐2‐interacting protein 1 (Beclin‐1) and inducing autophagy. Based on the above, we speculate the pathway of HIF‐1α/miR‐20a exists and acts in MIRI by regulating autophagy [22]. But very few reports on it have been extensively studied.

Apigenin (API), a flavonoid, is widely distributed in vegetables and fruits in warm zones in nature, so it is named because of its highest content in celery [23]. It is found that API can protect rat myocardium from injury by inhibiting oxidative stress and myocardial cell necrosis, increasing levels of autophagy‐related genes, and then restoring left ventricular function [24]. The above studies suggested the potential effects of API in anti‐MIRI therapy by regulating autophagy. Therefore, in this study we aimed at elucidating the anti‐MIRI effect of API and exploring the specific molecular mechanism. Using the established H9c2 OGD/R cell model, we hypothesized that API could alleviate MIRI by inhibiting autophagy via the HIF‐1α/miR‐20a pathway. This study may provide new insights into the roles of API in MIRI therapy.

## 2 Material and Methods

### 2.1 Drugs and Reagents

API (HPLC ≥ 98% CAS:520‐36‐5) was purchased from Shanghai Yuanye Bio‐Technology Co. Ltd. (Shanghai, China). When used, it is dissolved in dimethyl sulfoxide (DMSO) at 100 mM concentrations. MTT was purchased from Abcam (UK). HIF‐1α inhibitor Guanylyl cyclase activator (YC‐1) (BIOFOUNT, China), autophagy inhibitor CA‐5f, and autophagy activator Rapamycin (RAPA) (Med Chem Express, China) were dissolved in DMSO at 10 μM and 100 nM, respectively.

### 2.2 Molecular Docking

In the PubChem database (https://pubchem.ncbi.nlm.nih.gov/), we retrieved about “apigenin” to choose the “BEST MATCH” result, downloading “2D Structure, SDF” style, and used “Open Babel GUI” software to convert “SDF” to “PDB” style. In the Uniprot database (https://www.uniprot.org/), we retrieved the abbreviation of target proteins and chose the “Reviewed, Human, BEST MATCH” result. In the “Structure” title, “SOURCE” and “METHOD” chose “PDB” and “X‐ray” as the filter criteria respectively, and the result with the lowest RESOLUTION value is favorite in “RESOLUTION”. Leading the “PDB ID” number to the PDB database (https://www.rcsb.org/), we downloaded its “PDB Format” style after verifying “Mutation(s): 0”. The PDB ID numbers were: HIF‐1α(1H2K), ATG5(4TQ1), ATG16L1(4GDL), Beclin1(4DDP), ULK1(6HYO). The solvent, organic, and residues were removed from target proteins in the Pymol.exe software, and API was docked with target proteins in AutoDock Tools 1.5.6 software. The minimum binding energy between the API and the target proteins was obtained, using minimum binding energy to estimate the binding affinity. Finally, the file of minimum binding energy was visualized in the Pymol.exe software.

### 2.3 Cell Culture and Cardiomyocyte Model of OGD/R

The rat H9c2 (RRID: CVCL_0286) cardiomyocyte cell line was purchased from the Shanghai Cell Bank of the Chinese Academy of Sciences (Shanghai, China), Cat. No. SCSP‐5211. Cell quality control test (test report available): (1) Mycoplasma test result: negative; (2) Bacterial test result: negative; (3) Fungal test result: negative; (4) Species identification result: The cell is a rat cell. The H9c2 cells were cultured in Dulbecco's modified Eagle's medium (DMEM) (Biological Industries, Israel) supplemented with 10% fetal bovine serum (FBS) (CellMax, Australia), 1% penicillin (100 U/mL), and 1% streptomycin (100 *μ*g/mL) in an incubator (Thermo Fisher Scientific, USA) at 37°C in a humidified atmosphere of 21% O_2_ and 5% CO_2_. The experimental methods related to apigenin are as follows: pretreatment time: We first incubate H9c2 cells with different concentrations of apigenin for 24 h. Administration time during OGD/R injury: a total of 20 h, including 16 h of glucose oxygen deprivation and 4 h of reoxygenation (during this stage, the API continues to exist in the culture system). The specific modeling method of OGD/R model refers to the previous work of our group [25].

### 2.4 Cell Viability Assay

After cell transfection or treated with API or specific inhibitors and OGD/R treatment, H9c2 cells were seeded into 96‐well plates (9 × 10^3^ cells per well) and cultured in a 5% CO_2_ incubator at 37°C. After that, 100 μL of MTT reagent was added to each well and maintained in the same incubator for 4 h. Thereafter, a microplate reader (BMG Labtech, Oefenburg, Germany) was used for measurement of the absorbance at 570 nm.

### 2.5 Cell Transfection

MiR‐20a inhibitor and mimics and its corresponding control (miR‐NC) were purchased from GenePharma. A transient transfection was performed using Lipofectamine 3000 (Thermo Fisher Scientific, Waltham, MA, USA). In brief, 48 h prior to transfection, 5 × 10^5^ H9c2 cells were plated in a six‐well plate. Cells were transfected with 60 nM miR inhibitor. After 48 h, cells were subjected to total RNA extraction for RT‐qPCR detection.

### 2.6 Cellular Injury Assessment

After OGD/R inducement and treated with API or specific inhibitors and cell transfection, the productions of Lactate dehydrogenase (LDH) and superoxide dismutase (SOD) were measured using SOD assay kit (A001‐3‐2, Nangjing Jiancheng Bioengineering institute) and LDH assay kit (A020‐2‐2, Nangjing Jiancheng Bioengineering institute). The productions of MDA and GSH were measured using MDA assay kit (A003‐4‐1, Nangjing Jiancheng Bioengineering institute) and GSH assay kit (A006‐2‐1, Nangjing Jiancheng Bioengineering institute).

### 2.7 ROS Assay

After seeding cells into confocal dishes and subjecting them to different treatments for the corresponding time points, add 1 mL of DCFH‐DA (1×) diluted in serum‐free medium to each well, followed by incubation in a cell incubator for 30 min. Upon completion of incubation, wash the cells three times with serum‐free cell culture medium to fully remove the DCFH‐DA that has not penetrated into the cells. Then add Hoechst 33258 (1×) to each well, continue incubating the cells in the cell incubator for 10 min, and subsequently take out the cells and wash them twice with serum‐free medium. Finally, add 1 mL of serum‐free medium and image the cells using a fluorescence microscope.

### 2.8 Quantitative Reverse Transcription Polymerase Chain Reaction (RT‐qPCR)

Trizol reagent (Solarbio, Beijing, China) was used for the isolation of total RNAs with the existence of DNase I (Sangon Biotech, Shanghai, China). The Nanodrop 2000 system (Thermo Fisher Scientific) was employed for measurement of the concentration of isolated RNAs. Synthesis of the first strand of cDNA was completed with the help of Hifair V one‐step RT‐gDNA digestion SuperMix for qPCR (Yeasen, Shanghai, China). After that, qRT‐PCR was carried out by using Hieff UNICON Universal Blue qPCR SYBR Green Master Mix (Yeasen). The primers used were as follows (Table 1).

**TABLE 1:** Primer details.

Primer name	Forward sequence (5′ → 3′)	Reverse sequence (5′ → 3′)
HIF‐1α	5′‐GCG GCG AGA ACG AGA AGAA‐3′	5′‐GCC ATC CAG TCC GGC TTT CAG AT‐3′
ATG16L1	5′‐CAA GCC GAA TCT GGA CTG TGG ATG‐3′	5′‐CAG CAG GAA CTT GGC AGA GAG AAC‐3′
Beclin1	5′‐CAG TGG CGG CTC CTA TTC CA‐3′	5′‐AGG ACA CCC AAG CAA GAC CC‐3′
ULK1	5′‐TAC ACA GCA AGG GCA TCA TTC ACC‐3′	5′‐CGG GCA AAT CCA AAG TCA GCA ATC‐3′
β‐actin	5′‐GTG GAC ATC CGC AAA GA CAC‐3′	5′‐GCT GTC ACC TTC ACC GTTC‐3′
MiR‐20a	5′‐GCC GTA AAG TGC TTA TAG TGC AG‐3′	5′‐TAT GGT TTT GAC GAC AGT TGT GTG AT‐3′
U6	5′‐CTC GCT TCG GCA GCA CAT‐3′	5′‐TTT GCG TGT CAT CCT TGC G‐3′

The internal references for miR‐20a‐5p and HIF‐1α, ATG16L1, ULK1, Beclin1 were U6 and Actin, respectively. Gene expression was calculated by utilizing the 2^−△△Ct^ method.

### 2.9 Western Blot

Cellular protein extraction was conducted by using RIPA lysis buffer (BosterBio, California, USA) with the presence of a protease inhibitor (Sangon Biotech). BCA method (Solarbio) was employed for protein concentration determination. After completing sodium dodecyl sulfate polyacrylamide gel electrophoresis (SDS‐PAGE), serum albumin (BSA; AG Scientific Inc., San Diego, CA, USA) blocking was carried out on the membranes at room temperature for 2 h. Thereafter, primary antibody incubation was performed on the membranes at 4°C for 14 h. After rinsing, the HRP‐marked secondary antibody (Beijing, Soleibao Biotechnology Company) incubation was conducted at room temperature for 1 h. Primary antibodies were as follows: HIF‐1α (USA, Affinity Bioscience), Beclin1, ULK1, p62, LC3, β‐actin (Wuhan, Aibotech Biotechnology Co. Ltd). Data were normalized to β‐actin and were disposed of by ImageJ software.

### 2.10 Statistical Analysis

The data were expressed as the mean ± standard deviation (SD) from three independent experiments. Data analysis was performed by employing GraphPad Prism 9.0 software (GraphPad Prism Software Inc., USA). One‐way analysis of variance (ANOVA) was respectively employed for comparison among multi‐groups. Student's *t*‐test was used for comparison between two groups. *p* < 0.05 was acknowledged to be statistically significant.

## 3 Results

### 3.1 Molecular Docking

**FIGURE 1:**
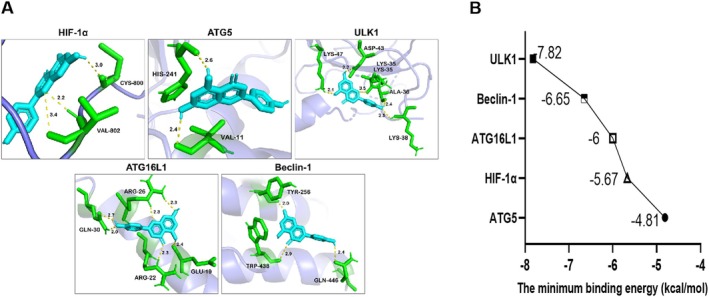
Molecular docking of API with HIF‐1α and autophagy‐related proteins. (A) Visualization of docking of apigenin with HIF‐1α and autophagy‐related proteins. (Gray purple is the target protein, turquoise is API, green is the club structure of amino acid residues, and yellow is hydrogen bond.) (B) Molecular docking binding energy.

Molecular docking technology was used to evaluate the binding affinity of API and target proteins. The docking results showed that API had a strong binding ability with HIF‐1α, ATG5, ATG16L1, Beclin1, and ULK1, with low binding energy values of −5.67, −4.81, −6, −6.65, and −7.82 kcal/mol, respectively (Figure 1) [23].

### 3.2 API Inhibited OGD/R‐Induced Myocardial Injury in H9c2 Cells

**FIGURE 2:**
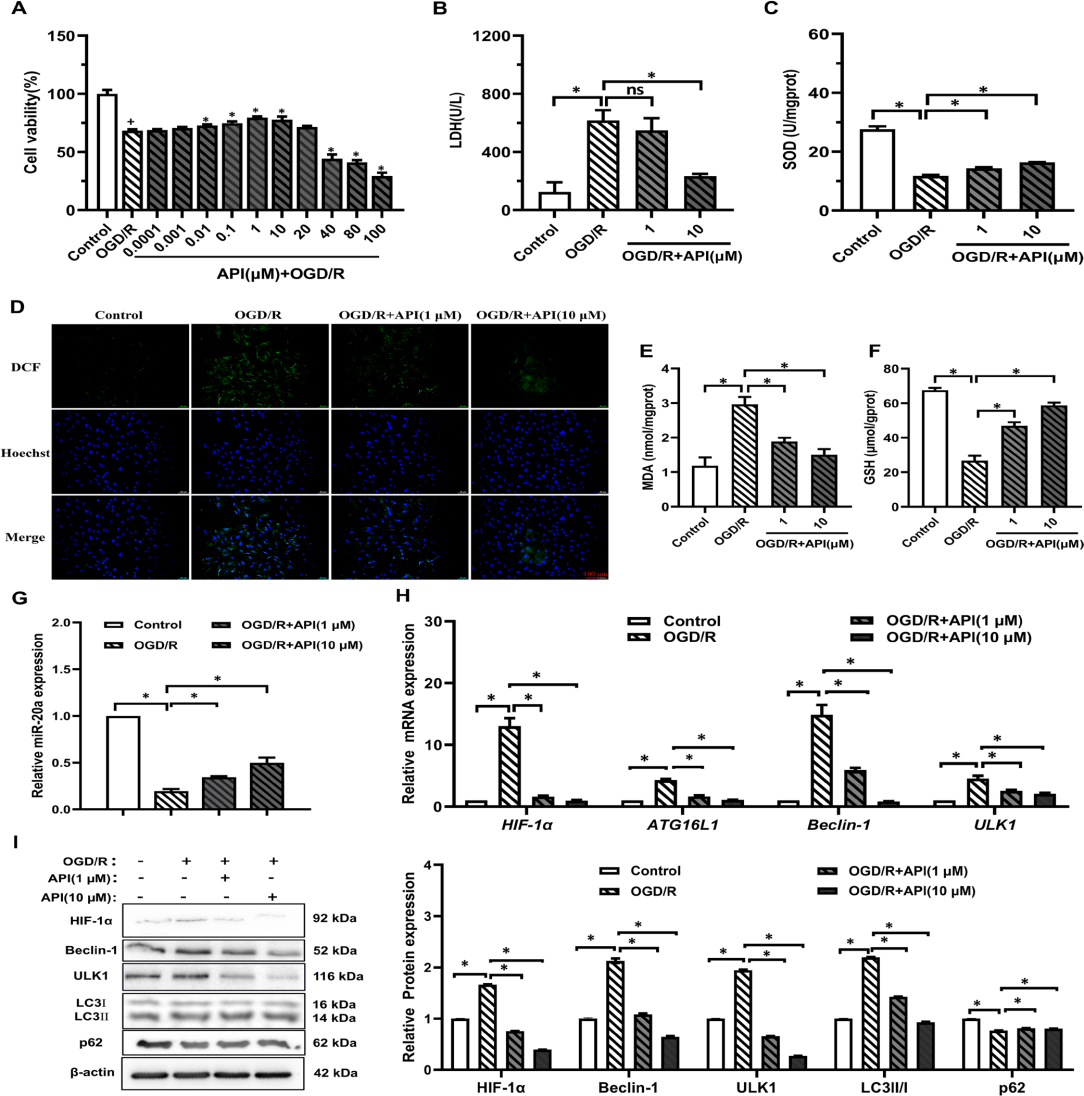
The effect of API on myocardial activity, SOD activity, LDH, MDA, GSH levels, and related molecular expression in H9c2 cells induced by OGD/R. (A) The viability of H9c2 cells treated with API (0.0001–100 μM) was assessed by MTT assay. (B, C) LDH leakage, SOD activity levels in H9c2 cells. (D) Cellular ROS levels after OGD/R and API application (scale bar: 100 μm). (E, F) Levels of MDA and GSH in different treatment groups. (G, H) The gene expression levels of miR‐20a, HIF‐1a and autophagy‐related proteins (ATG16L1, Beclin1, and ULK1) were detected by RT‐qPCR. (I) The expression of HIF‐1a and autophagy‐related proteins (Beclin1, ULK1, LC3II/I, p62) were detected by Western blot. *p < 0.05.

To investigate the effects of API on OGD/R‐induced myocardial injury in H9c2 cells, we conducted the following experiments. As illustrated in Figure 2A, cell viability of the OGD/R injury group was lower than that of the control group; however, treatment with API (0.01–10 μM), especially at 1 and 10 μM, significantly increased cell viability. No significant difference was observed between the OGD/R group and 20 μM API group. High concentrations of API (40 μM) decreased cell viability. We also investigated the protective effects of API at different concentrations with varying treatment durations on OGD/R‐induced cardiomyocytes (Figure S1). These results indicate that 0.01–10 μM API had the protective effect for the viability of H9c2 cells induced by OGD/R, but 40 μM API exhibited cytotoxicity effect. Thus, API at 1 and 10 μM were selected as the low and high doses, respectively, for the subsequent experiments.

As shown in Figure 2B, LDH levels increased in the OGD/R group compared to the control group, but decreased after treatment with 10 μM API The SOD data showed an opposite trend to the LDH results (Figure 2C). In Figure 2D, we used ROS probes to detect the ROS levels in cells after OGD/R, and also examined the effect of API on cellular ROS levels. The results showed that after OGD/R injury, green fluorescence was significantly enhanced and cellular ROS levels increased. Administration of API 1 and 10 μM could significantly reduce the increase in cellular ROS levels caused by OGD/R injury. After administration of API, the MDA level in cells was significantly reduced compared to the OGD/R group, and the GSH level was restored (Figure 2E,F). In Figure 2G, qRT‐PCR results showed an inhibition in miR‐20a mRNA expression in the OGD/R group compared to the control group, but 1 and 10 μM API could significantly reverse these results. In Figure 2H,I gene and protein levels of HIF‐1α, Beclin1, ULK1 increased in OGD/R group compared to the control group. Meanwhile, the WB results show that LC3II/I LC3 II/I ratio increases, yet the p62 protein expression decreased. After API treatment, HIF‐1α and the autophagy‐related protein levels (ATG16L1, Beclin1, ULK1, LC3II/I, p62) were reversed. Taken together, the above results basically indicate that 1 and 10 μM API inhibited OGD/R‐induced myocardial injury in H9c2 cells.

### 3.3 The Myocardial Cell Injury Induced by OGD/R Is Mediated by HIF‐1α/miR‐20a Axis

**FIGURE 3:**
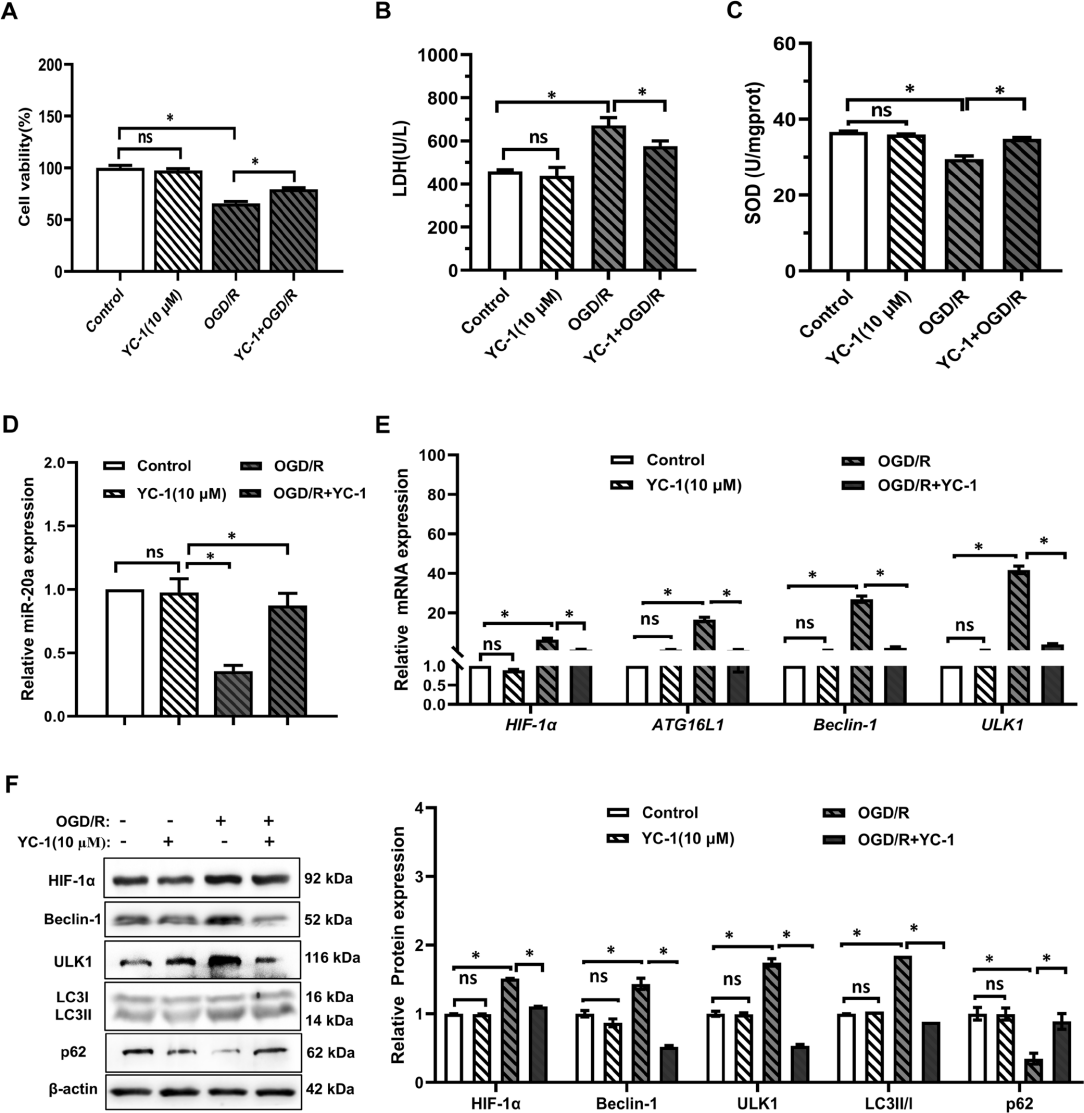
Effects of YC‐1 on OGD/R‐induced myocardial viability, SOD activity, LDH leakage, and related molecular expression in H9c2 cells. (A) The viability of H9c2 cells treated with YC‐1 (10 μM) was assessed by MTT assay. (B, C) LDH leakage, SOD activity levels in H9c2 cells. (D, E) The gene expression levels of miR‐20a, HIF‐1a and autophagy‐related proteins (ATG16L1, Beclin1, and ULK1) were detected by RT‐qPCR. (F) The expression of HIF‐1a and autophagy‐related proteins (Beclin1, ULK1, LC3II/I, p62) was detected by Western blot (*n* = 5). *p < 0.05.

To determine whether OGD/R‐induced myocardial cell injury is mediated by the HIF‐1α/miR‐20a axis, we used the HIF‐1α inhibitor YC‐1 and miR‐20a mimics, respectively. For YC‐1 intervention experiment, the OGD/*R* + YC‐1 group showed elevated cell viability and SOD levels, and a decline in LDH levels compared to the OGD/R group (Figure 3A–C). RT‐qPCR results indicated a decrease in the expression of HIF‐1α and autophagy‐related proteins (ATG16L1, Beclin1, ULK1) in the OGD/*R* + YC‐1 group, but an increase in the expression of miR‐20a compared to the OGD/R group (Figure 3D,E). The Western blot results indicate that the expression of HIF‐1α, Beclin1, ULK1, and LC3II/I protein decreased in the OGD/*R* + YC‐1 group compared to the OGD/R group. Conversely, the expression of p62 protein increased (Figure 3F). These results suggest that blocking HIF‐1α may prevent OGD/R‐induced myocardial cell injury by regulation of miR‐20a, oxidative stress, and autophagy.

**FIGURE 4:**
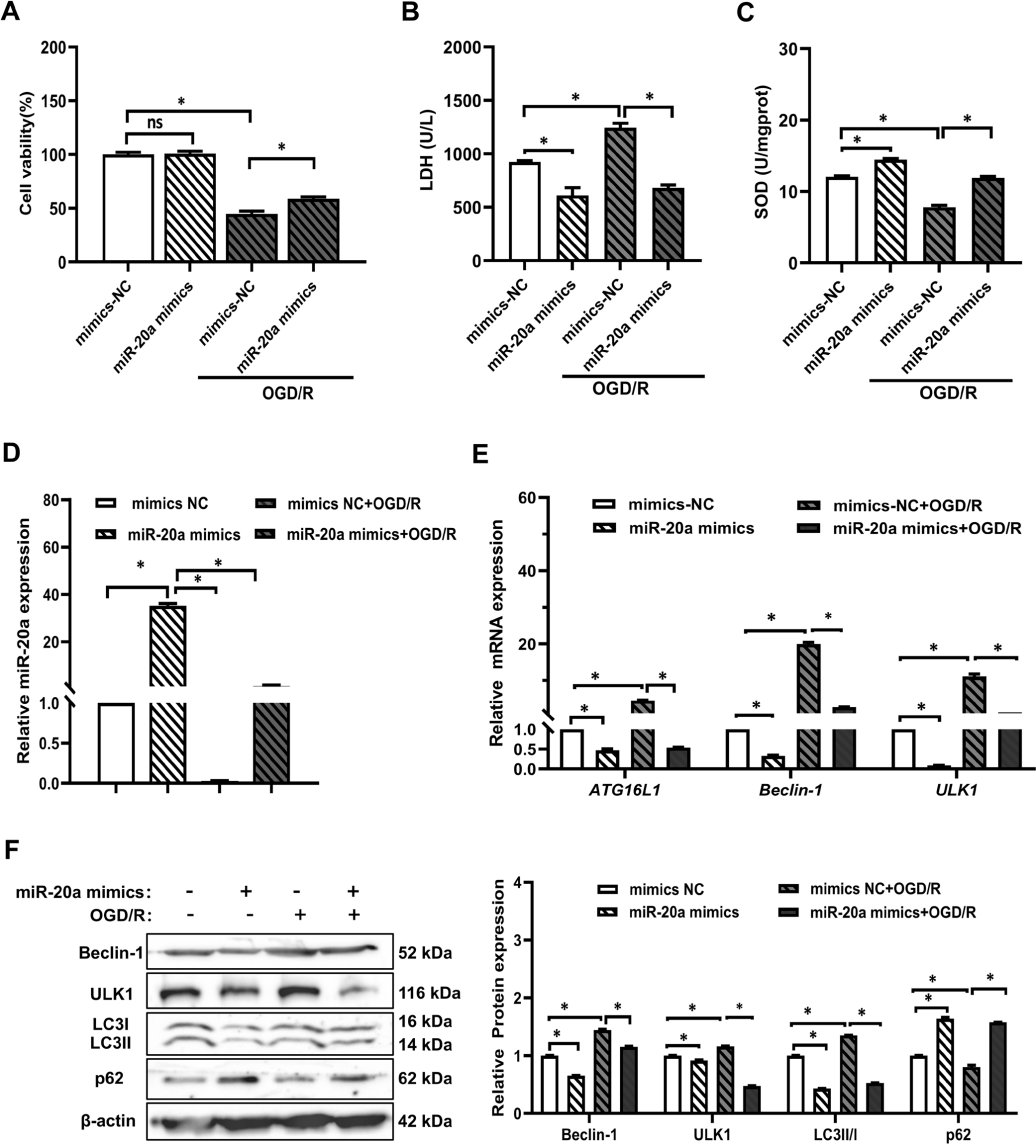
Effects of miR‐20a mimics on OGD/R‐induced viability, SOD activity, LDH leakage, expression of miR‐20a and autophagy‐related molecules in H9c2 cells. (A) The viability of H9c2 cells treated with miR‐20a mimics (60 nM) was assessed by MTT assay. (B, C) LDH leakage, SOD activity levels in H9c2 cells. (D, E) The gene expression levels of miR‐20a and autophagy‐related proteins (ATG16L1, Beclin1, and ULK1) were detected by RT‐qPCR. (F) The expression of autophagy‐related proteins (Beclin1, ULK1, LC3II/I, p62) was detected by Western blot. (*n* = 5). *p < 0.05.

For miR‐20a mimics experiment, OGD/*R* + miR‐20a mimics group showed elevated cell viability and SOD levels, and a decline in LDH levels compared to the OGD/*R* + mimics NC group (Figure 4A–C). RT‐qPCR results revealed an increase in miR‐20a expression in the OGD/*R* + miR‐20a mimics group compared to the OGD/*R* + mimics NC group, while the expression of autophagy‐related proteins (ATG16L1, Beclin1, ULK1) decreased (Figure 4D,E). The Western blot results indicate that the expression of Beclin1, ULK1 and LC3II/I protein decreased in the OGD/*R* + miR‐20a mimics group compared to the OGD/R + mimics NC group. Additionally, the expression of p62 protein increased (Figure 4F). These results suggest that miR‐20a, the target gene regulated by HIF‐1α, mediates OGD/R‐induced myocardial cell injury and negatively regulates the oxidative stress and autophagy.

### 3.4 The Myocardial Cell Injury Induced by OGD/R Is Mediated by Autophagy

**FIGURE 5:**
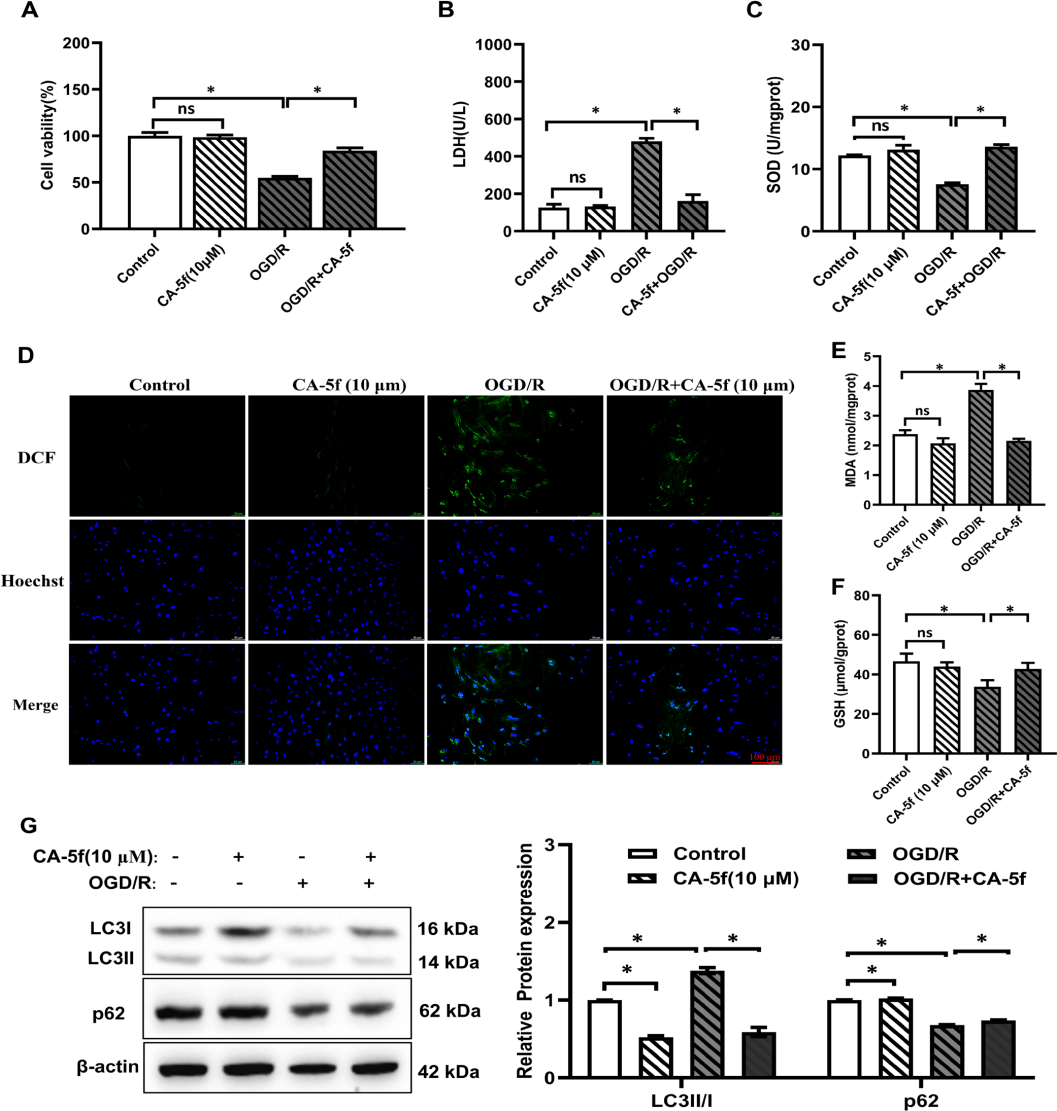
Effects of CA‐5f on OGD/R‐induced viability, SOD activity, LDH, MDA, GSH levels, and autophagy marker protein expression in H9c2 cells. (A) The viability of H9c2 cells treated with CA‐5f (10 μM) was assessed by MTT assay. (B, C) LDH leakage, SOD activity levels in H9c2 cells. (D) Cellular ROS levels after OGD/R and CA‐5f (10 μM) application (scale bar: 100 μm). (E, F) Levels of MDA and GSH in different treatment groups. (G) The expression of autophagy‐related proteins (LC3II/I, p62) was detected by Western blot. (*n* = 5). *p < 0.05.

Next, we used the autophagy inhibitor CA‐5f to verify whether OGD/R‐induced myocardial cell injury is mainly mediated by autophagy. The OGD/*R* + CA‐5f group showed elevated cell viability and SOD levels, and a decline in LDH levels compared to the OGD/R group (Figure 5A–C). Compared with the OGD/R group, the OGD/*R* + CA‐5f group exhibited a decrease in cellular ROS levels (Figure 5D). Compared with the OGD/R group, the OGD/*R* + CA‐5f group showed a significant decrease in MDA levels and an increase in GSH levels (Figure 5E,F). Western blot results indicated a decrease in the expression of LC3II/I protein and an increase in the expression of p62 protein in the OGD/*R* + CA‐5f group compared to the OGD/R group (Figure 5G). Therefore, autophagy mediated the OGD/R‐induced cell injury by increasing oxidative stress.

### 3.5 API Reduces OGD/R‐Induced Myocardial Cell Injury by Regulating miR‐20a

**FIGURE 6:**
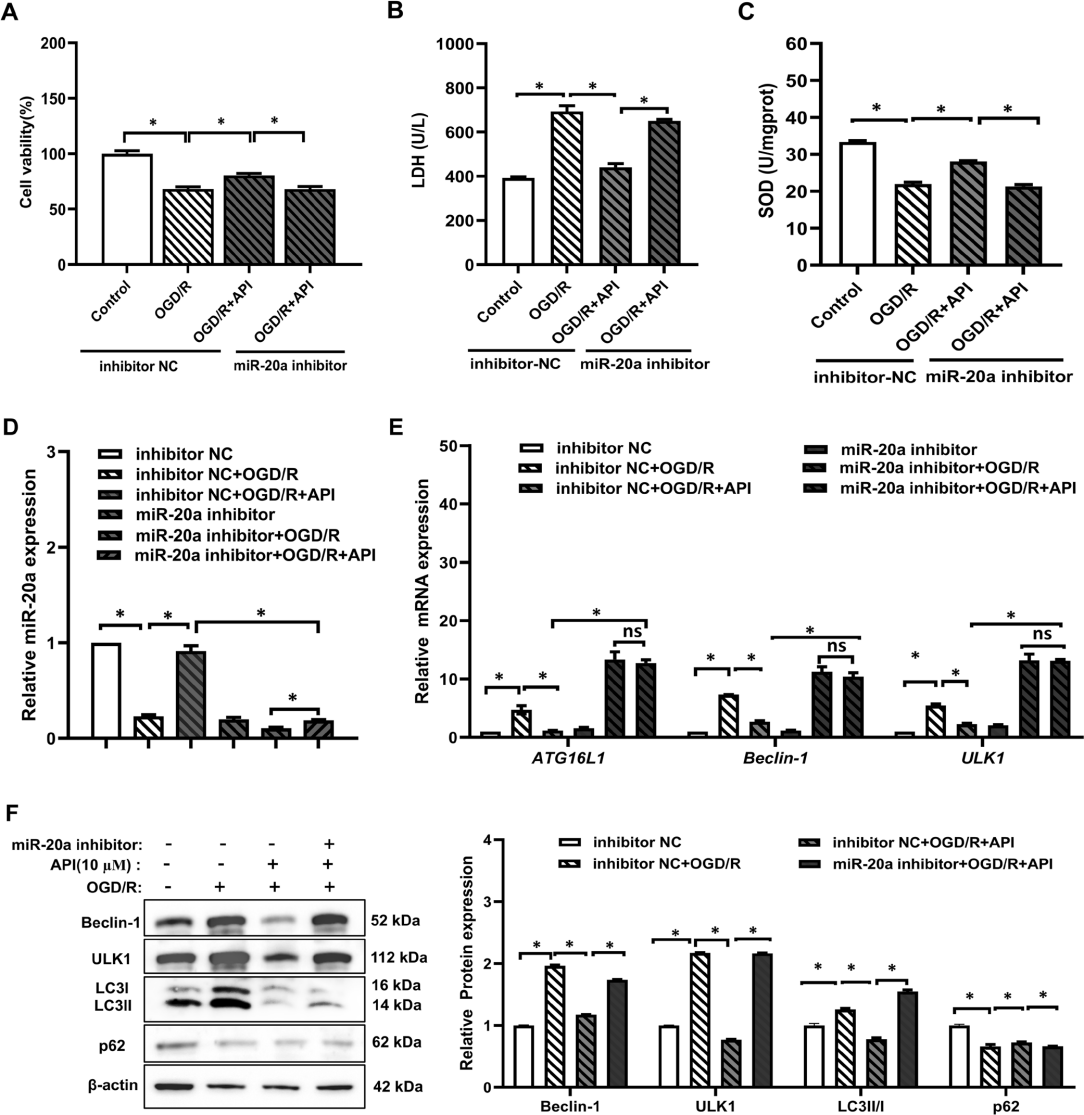
Effects of miR‐20a inhibitor on cell viability, SOD activity, LDH leakage, and expression of miR‐20a and autophagy‐related molecules in API‐treated H9c2 cells. (A) The viability of H9c2 cells treated with miR‐20a inhibitor (60 nM) was assessed by MTT assay. (B, C) LDH leakage, SOD activity levels in H9c2 cells. (D, E) The gene expression levels of miR‐20a and autophagy‐related proteins (ATG16L1, Beclin1, and ULK1) were detected by RT‐qPCR. (F) The expression of autophagy‐related proteins (Beclin1, ULK1, LC3II/I, p62) was detected by Western blot. (*n* = 5). *p < 0.05.

To investigate whether API alleviates myocardial cell damage in the OGD/R model via miR‐20a, we employed a miR‐20a inhibitor. After inhibiting miR‐20a, the miR‐20a inhibitor + OGD/*R* + API group showed decreased cell viability (Figure 6A), increased LDH levels (Figure 6B), and decreased SOD levels (Figure 6C) compared with the inhibitor NC + OGD/*R* + API group. These results suggest that the protective effect of API on cells depends on miR‐20a. Additionally, we observed that API promotes miR‐20a expression in cells (Figure 6D). Following API application, there was significant suppression of the mRNA levels of the autophagy‐related genes ATG16L1, Beclin1, and ULK1 (Figure 6E). Western blot (WB) results showed that, after applying the miR‐20a inhibitor, the expression of the autophagy‐related proteins Beclin1 and ULK1 increased; the LC3 II/I ratio rose; and P62 protein expression decreased (Figure 6F). These results suggest an increase in autophagy levels. These results suggest that API alleviates myocardial cell damage in the OGD/R model by promoting miR‐20a expression, which inhibits oxidative stress and autophagy.

### 3.6 API Reduces OGD/R‐Induced Myocardial Cell Injury by Regulating Autophagy

**FIGURE 7:**
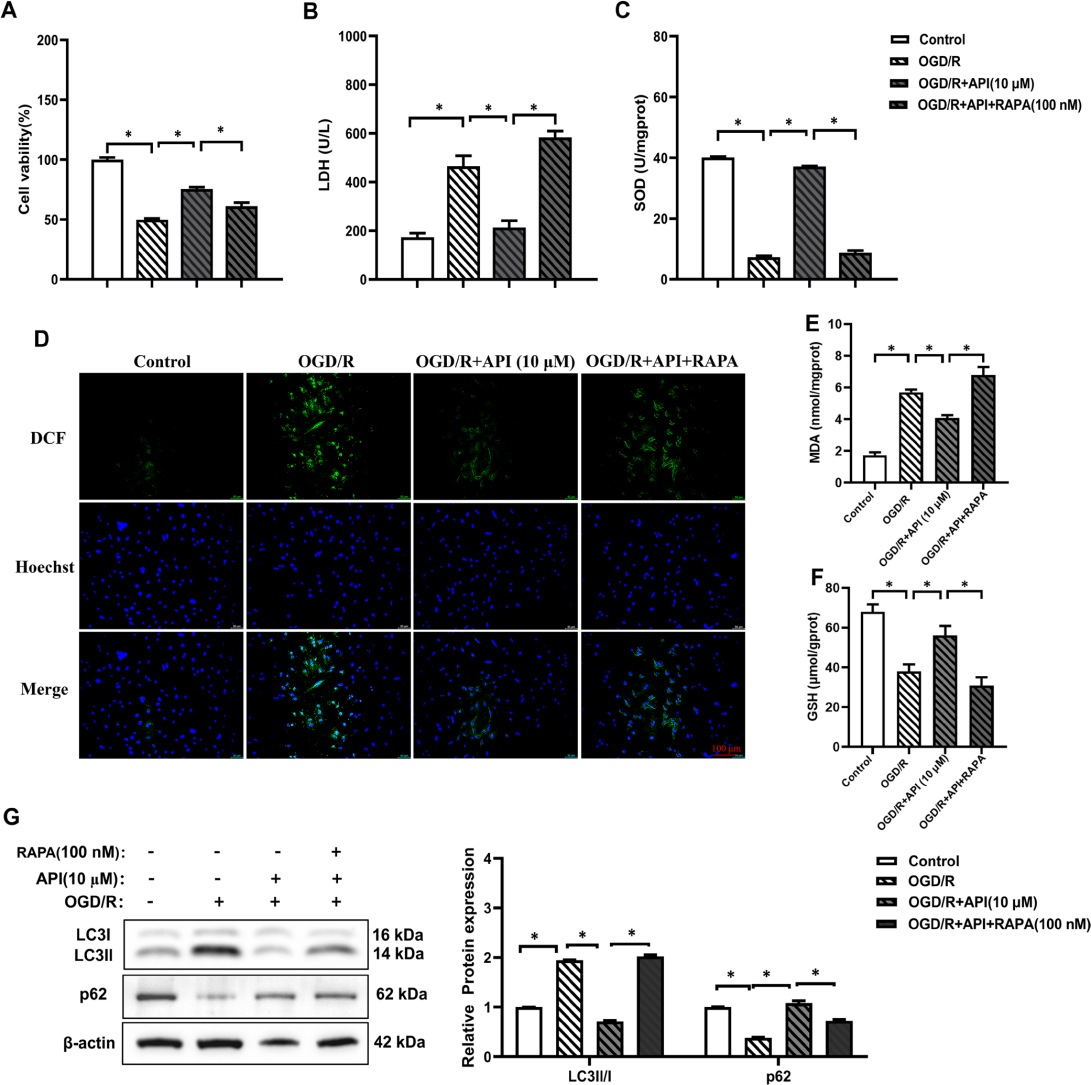
Effects of Rapamycin on cell viability, SOD activity, LDH, MDA, GSH levels, and autophagy marker protein expression in API‐treated H9c2 cells. (A) The viability of H9c2 cells treated with API (10 μM) and RAPA (100 nM) was assessed by MTT assay. (B, C) LDH leakage, SOD activity levels in H9c2 cells. (D) Cellular ROS levels after OGD/R and API (10 μM), RAPA (100 nM) application (scale bar: 100 μm). (E, F) Levels of MDA and GSH in different treatment groups. (G) The expression of autophagy‐related proteins (LC3II/I, p62) was detected by Western blot (*n* = 5). *p < 0.05.

To investigate whether API alleviates myocardial cell injury via inhibiting autophagy in the OGD/R model, the autophagy activator rapamycin (RAPA) was used. Compared with the OGD/*R* + API group, the OGD/*R* + API + RAPA group showed decreased cell viability (Figure 7A), increased lactate dehydrogenase (LDH) levels (Figure 7B), and decreased superoxide dismutase (SOD) levels (Figure 7C) after adding RAPA. Compared with the OGD/*R* + API group, the addition of RAPA significantly increased the cellular ROS levels in the OGD/*R* + API + RAPA group (Figure 7D). At the same time, after adding RAPA, the MDA level in the OGD/R + API + RAPA group increased while the GSH level decreased (Figure 7E,F). These results indicate that the protective effect of API on cell viability is partially attributed to its inhibition of autophagic levels in cells after OGD/R. As shown in Figure 7G, compared with the OGD/*R* + API (10 μM) group, the OGD/R + API + RAPA group exhibited an increased LC3II/I ratio and decreased p62 protein expression, further confirming that API alleviates myocardial cell injury in the OGD/R model by inhibiting autophagy.

## 4 Discussion

**FIGURE 8:**
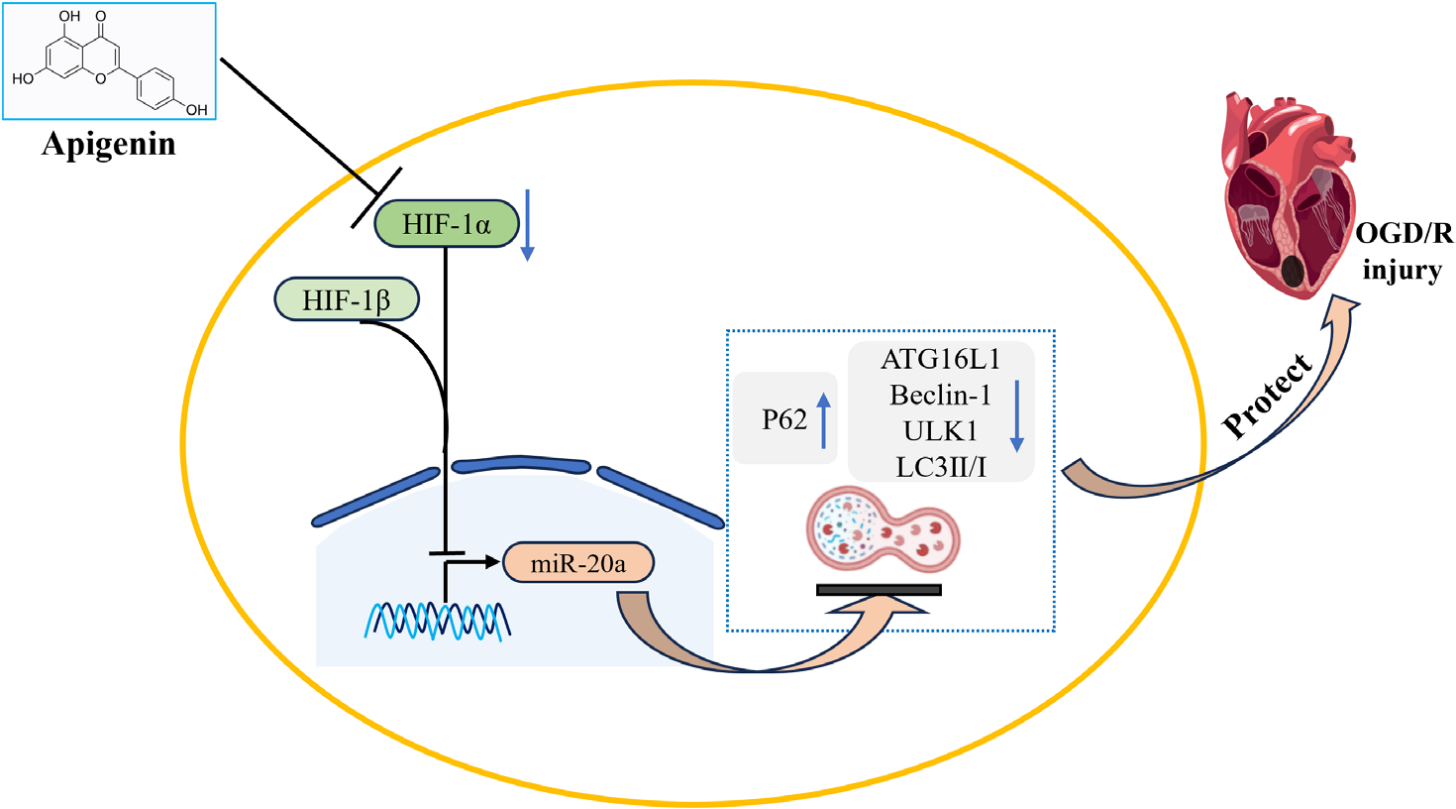
Mechanism diagram of apigenin protecting OGD/R‐induced damage to H9c2 cardiomyocytes.

The OGD/R‐induced myocardial cell injury serves as an in vitro model for studying drug intervention in MIRI. In this study, we used the H9c2 OGD/R cell model established before to confirm that API regulates autophagy through the HIF‐1α/miR‐20a loop and plays a protective role in OGD/R‐induced myocardial cell injury (Figure 8). API plays the potential therapeutic role in MIRI clinical intervention.

Acute myocardial infarction (AMI) is the primary cause of cardiovascular death [26]. Recent studies have reported the significance of autophagy in the pathogenesis of AMI [27, 28]. Autophagy is a cellular housekeeping process that can either promote cell survival or lead to cell death, depending on the cellular context [29, 30]. When cells experience nutrient starvation, energy deprivation, oxidative stress, or ER stress, autophagy is activated as a pro‐survival response [31]. However, excessive or prolonged insult can cause severe and harmful upregulation of autophagy, ultimately resulting in cellular death [32]. Studies have shown that the clearance rate of autophagy is impaired during I/R injury, leading to autophagy acceleration and myocardial cell death [33]. Knocking out the Beclin1 heterozygote significantly reduces I/R‐induced autophagy formation, accompanied by decreased apoptosis and myocardial infarction [34]. Similarly, inhibiting autophagy by 3‐methyladenine or Beclin1 siRNA can reduce I/R‐induced autophagy and promote cell survival [35].

In the present study, we observed the enhanced autophagy with the increased expression of HIF‐1α in H9c2 cells induced by OGD/R. Inhibition of HIF‐1α reduced autophagy and autophagy‐related proteins, including ATG16L1, Beclin1, ULK1, and LC3II/I. HIF‐1α is a hypoxia‐inducing factor that contributes to the development of cardiovascular diseases such as myocardial ischemia, myocardial infarction, and heart failure [36]. Studies have shown that under oxygen and glucose deprivation, upregulation of HIF‐1α expression in microglia can induce autophagy, promote cell death, and lead to the secretion of inflammatory factors [37]. Simultaneously, the use of siRNA technology (The full name of siRNA is small interfering RNA, which is a double‐stranded RNA molecule with a length of 20 to 25 nucleotides and has a specific structure and function. SiRNA mainly regulates gene expression through RNA interference (RNAi) mechanism.) to interfere with HIF‐1α expression can inhibit autophagy and cell death of microglia [38]. The above studies including our results demonstrated that upregulation of HIF‐1α promotes OGD/R‐induced autophagy of H9c2 cells and exacerbates cell damage.

Researchers reported that inhibition of miR‐20a activated autophagy flux by up‐regulating the expression of autophagy‐related proteins including BECN1, ATG16L1, and SQSTM1/p62 [39]. Conversely, over‐expression of miR‐20a can inhibit autophagy and lysosomal proteolytic activity by down‐regulating them [40]. MiR‐20a expresses lowly in cardiovascular diseases such as acute myocardial infarction, serving as a diagnostic biomarker for coronary artery disease [41]. Ten years ago, miR‐20a was designated as an autophagy‐related gene for the first time, as it can negatively regulate autophagy of C2C12 myoblasts [42]. Under hypoxia, HIF‐1α inhibits miR‐20a, which negatively regulates the autophagy‐related gene ATG16L1. This consolidates the regulatory axis of HIF1A‐miRNA‐20a‐ATG16L1 as the key mechanism of hypoxia‐induced autophagy in osteoclast differentiation [21]. In the present study, we found that in H9c2 cells induced by OGD/R, miR‐20a was down‐regulated and autophagy‐related protein expression was up‐regulated. Besides, activating HIF‐1α down‐regulated miR‐20a expression and enhanced autophagy. Therefore, our findings suggest that miR‐20a acts as the downstream target of HIF‐1α, resulting in the up‐regulation of its endogenous target autophagy‐related protein activity. Furthermore, we used the autophagy inhibitor CA‐5f to testify that the OGD/R cardiomyocytes' injury was induced by autophagy via the HIF‐1α/miR‐20a axis.

Modern pharmacological research shows that API has many pharmacological effects, such as anti‐depression, anti‐inflammation, anti‐tumor, anti‐oxidation, anti‐bacteria, anti‐injury, anti‐aging, and anti‐obesity. It is a natural active ingredient with diverse curative effects, safety, and low toxicity [43]. Wang et al. found that API could improve myocardial cell injury by decreasing oxidative stress and increasing levels of autophagy‐related genes such as *Mtor* [44]. Our study proved that API could reduce the OGD/R damage of H9c2 cells by inhibition of oxidative stress and autophagy through up‐regulating the miR‐20a pathway. Further results using the autophagy activator RAPA demonstrated that API directly regulated autophagy to alleviate myocardial cell injury in the OGD/R model.

The H9c2 cell line, as an in vitro model for studying myocardial ischemia–reperfusion (I/R) injury, has certain translational potential and limitations. H9c2 cells can simulate I/R injury through hypoxia/reoxygenation (H/R) or oxygen–glucose deprivation/reperfusion (OGD/R) with high operational reproducibility [45], and are suitable for investigating molecular mechanisms such as oxidative stress (e.g., malondialdehyde (MDA) and reactive oxygen species [ROS] detection), apoptosis (terminal deoxynucleotidyl transferase dUTP nick‐end labeling [TUNEL] assay and caspase‐3 activity measurement), and autophagy (LC3‐II/Beclin‐1 expression analysis) [46, 47, 48]. As a rat embryonic cardiomyocyte line, H9c2 lacks the three‐dimensional structure and electrophysiological characteristics of mature cardiomyocytes, which may underestimate the complexity of in vivo I/R injury [49]. Additionally, H9c2 is derived from rat embryonic myocardium, exhibiting species‐specific differences in gene expression compared to human cardiomyocytes [50].

## 5 Conclusion

This study confirms that apigenin (API) exerts a protective effect against oxygen–glucose deprivation/reperfusion (OGD/R)‐induced injury in H9c2 cardiomyocytes. Results show that 1–10 μM API effectively improves cell viability, reduces LDH leakage, and mitigates oxidative stress by lowering ROS and MDA levels while restoring SOD and GSH activity. Mechanistically, API functions by inhibiting the HIF‐1α/miR‐20a signaling axis: it downregulates HIF‐1α expression to upregulate miR‐20a, thereby suppressing autophagy‐related proteins and excessive autophagy, which is verified by relevant inhibitors and activators. However, this study has limitations: the H9c2 cell model lacks mature cardiomyocyte characteristics, and Western blot results lack validation by immunofluorescence or immunohistochemistry (IF/IHC), which also serves as a key direction for subsequent research. Collectively, our findings identify API as a promising natural candidate for treating myocardial ischemia–reperfusion injury via the HIF‐1α/miR‐20a‐autophagy pathway.

## Author Contributions

**Xiaoxu Zhao:** methodology, validation, formal analysis, investigation, data curation, writing – original draft preparation. **Huihui Li:** reviewing and editing, visualization, data curation. **Yi Yuan:** methodology, validation, formal analysis, investigation. **Qingwen Cao:** reviewing and editing, visualization, data curation. **Bo Yu:** validation, formal analysis. **Wenshu Xue:** methodology, validation. **Gongping Yu:** formal analysis, data curation. **Yao Wang:** formal analysis, data curation, funding acquisition. **Xiulong Niu:** methodology, formal analysis, funding acquisition. **Yue Wang:** formal analysis, data curation, writing – reviewing and editing, supervision.

## Funding

This study is funded by the following projects. The Independent innovation research projects of Characteristic Medical Center of PAP (KYZZCX2401). Tianjin Health Research Project (TJWJ2023MS050). National Natural Science Foundation of China (82104671). Tianjin University of Traditional Chinese Medicine Talented Youth Program (XJS2023108). Graduate Research Innovation Fund of Tianjin University of Traditional Chinese Medicine (ZXYCXLX202119).

## Ethics Statement

The authors have nothing to report.

## Conflicts of Interest

The authors declare no conflicts of interest.

## Supporting information


**Figure S1:** prp270224‐sup‐0001‐FigureS1.docx.

## Data Availability

The data that support the findings of this study are available from the corresponding author upon reasonable request.
