# Supplementary material for: Apigenin Protects H9c2 Cells Against Oxygen–Glucose Deprivation/Reperfusion Injury by Regulating Autophagy via the HIF‐1α/miR‐20a Axis
Source: Pharmacol Res Perspect. 2026 Mar 30;14(2):e70224. doi: 10.1002/prp2.70224 (PMC13042881; doi:10.1002/prp2.70224)
Supplement: Supplementary file 1 — Figure S1: prp270224‐sup‐0001‐FigureS1.docx. [file PRP2-14-e70224-s001.docx]

**Fig.S1 Protective effects of different concentrations of API treated for different durations on OGD/R cells**

*: *P*<0.05, compared with OGD/R group; +: *P* <0.05 compared with control group.
